# Reshaping cortical connectivity in traumatic spinal cord injury: a novel effect of hyperbaric oxygen therapy

**DOI:** 10.1038/s41394-021-00441-2

**Published:** 2021-09-09

**Authors:** Francesco Marrosu, Stefano Mancosu, Gianluca Lai, Matteo Fraschini, Antonella Muroni, Matteo Demuru, Marco Monticone, Giovanni Defazio

**Affiliations:** 1 AIAS Cagliari, Cagliari, Italy; 2 Intensive Care and Centre of Hyperbaric Medicine, ATS Sardegna, Cagliari, Italy; 3 Department of Electric and Electronic Engineering, University of Cagliari, Cagliari, Italy; 4 Dipartimento di Scienze Mediche e Sanita’ Pubblica, University of Cagliari, Cagliari, Italy

**Keywords:** Neuroscience, Medical research

## Abstract

**Introduction:**

Spinal cord injuries (SCIs) represent a severe neuro-traumatic occurrence and an excruciating social burden. Though the hyperbaric oxygen (HBO2) has been credited as a first line therapeutic resource for SCIs, its mechanism of action in the spine is only partially known, while the impingement upon other areas of the nervous system deserves additional investigation. In this study we deem to describe a novel effect of HBO2 in a subject affected by SCI who, along with the clinical improvement, showed a reshaped connectivity in cortical sensory-motor areas.

**Case presentation:**

A 45 years male presenting severe sensory-motor symptoms following a spinal lesion partially involving the C1 segment was successfully treated with HBO2 cycles. After the dramatic improvement reflected by an excellent optimization of the single performances, it has been investigated whether this result would reveal not only an intrinsic effect upon the spinal cord, but also a better connectivity strength in sensory-motor cortical regions. The results obtained by implementing EEG recordings with EEGLAB auto regressive vector plugins indeed suggest a substantial reshaping of cortico-cortical connectivity after HBO2.

**Discussion:**

These results show a correlation between positive clinical evolution and a new modulation of cortical connectivity. Though further clinical investigations would clarify as to whether HBO2 might be directly or epiphenomenally involved in this aspect of the network architecture, our report suggests that a comparison between clinical results and the study of brain connectivity represent a holistic approach in investigating the physiopathology of SCIs and in monitoring the treatment.

## Introduction

Although the hyperbaric oxygen (HBO2) treatment introduced a new hope in the therapy of spinal cord injuries (SCIs) [1], its mechanism of action remains still unclear [2]. This difficult can be explained by the fact that, despite the frequency of SCIs [3], the manifold anatomo-functional circuitries of the spinal cord are also differently affected. Indeed, variations in the localization and extension of SCIs, as usually reported in traumatic events, give rise to clinical expressions seldom acquiring a sufficient statistical power in their clinical occurrence that cannot fit into a unique strategy of neuropathological investigation [4]. Though these findings suggest a different impingement of HBO2 upon the lesioned areas, nonetheless these differences become an opportunity to inquire peculiar aspects of SCIs physiopathology. Although the “connectomic” has recently shed light on the role of intra and intercortical connections as it highlights new aspects of brain dynamics [5], to the best of our knowledge the interest in exploring the cortical connectivity in SCIs has been negligible. However, recent findings suggest a potential theoretical and pragmatic interest in investigating the relationships between possible new dynamics of brain connectivity and SCIs outcome of HBO2, given the premise that traumatic brain lesions increase the cortical neuroplasticity following HBO2 therapy [6]. By illustrating this case study we deem to investigate as to whether HBO2 impingement upon the putative neuronal micro scale dimensionality in the lesioned spinal cord would interfere with the meso-macroscale readout of the regional cortical connectivity as deduced by possible variations of brain EEG rhythms. Following the results obtained we suggest that the study of cortical connectivity could represent a potential new avenue of investigation of the HBO2 mechanism of action and a potential new tool in monitoring the effects of SCIs treatment.

## Case presentation

**Fig. 1:**
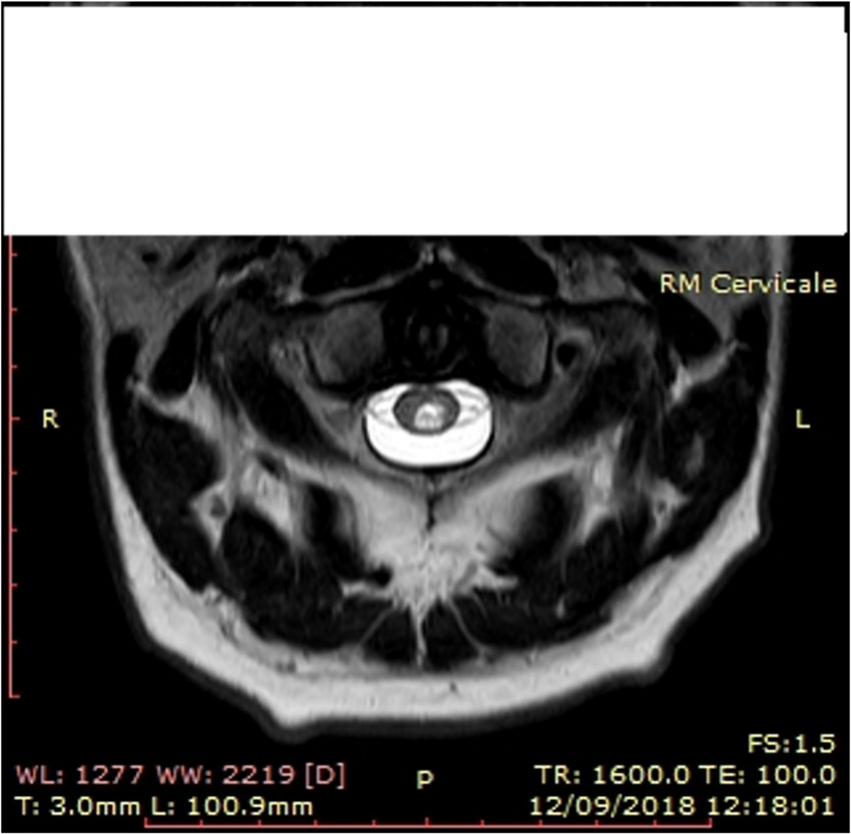
Cervical MRI. T1-weighted cervical MRI scan showing the spinal cord lesion in the medial posterior axial and sagittal C1 metamer.

A 45 years male, was admitted in the “Spinal Unit Division” of the Public Health Services of Cagliari (Italy), for a spinal lesion partially involving the C1 segment, caused by a dive. Familiar and personal anamnestic profile were unremarkable for pathologic events. The RM of the spinal cord showed a non hemorrhagic oedematous lesion localized in the C1 posterior part of the spinal cord (Fig. 1). According the International Standard for Neurological Classification of the Spinal Cord Injury [7] it has been ruled out a motor damage of the key muscles (level 5 present), while the sensory scoring revealed a severe (grade 1) impairment at level C1–C2. The clinical examination showed a modest unsteadiness, a bilateral severe apallesthesia, astereognosis and abataesthesia accompanied by a painful diffuse bilateral paresthesic feelings (referred as a mixed tingling-pricking and burning-numbness sensation) below C1 level. During the first 3 days following the trauma the subject was treated with low doses of methylprednisolone (AO Spine2016 https://aospine.aofoundation.org/Structure/Pages/default.aspx). No other drugs were administered during the present study. Moreover, in relation with the impairment of the discriminative sensitivity, eyes closure resulted in sudden falls, ataxia, motor incoordination and severe impairment of complex movements under command. After 3 months of rehabilitative program, as the static–dynamic equilibrium and the sensory-motor performances showed only a modest improvement, he underwent to a cycle of HBO2. Following the first cycle and despite that the neuroimages (MRI) acquired after the HBO2 therapy were unchanged compared to the initial features (not shown), the proprioceptive sensitivity as well as the static and dynamic motor performances resulted dramatically improved. However, as the HBO2 benefits lasted 6–8 weeks, new bimestrial cycles of HBO2 treatments were scheduled over time. Namely, in the follow up of 15 months the HBO2 therapy was repeated every 2 months. In details, the subject received five cycles of HBO2 treatment, each of which lasted 1 month of treatment (127 treatments administered in 18 sessions), spaced out by a HBO2 no treatment period of 2 months. The typical session of HBO2 treatment consisted in two periods of pure O2 administration at 2.5 absolute Atm. As the location of the hospital was at sea level, the administration of O2 is here intended at 1.5 Atm. Each period lasted 30 min, while in the 5 min of interval between the two periods the subject was allowed to breath ambient air given at the same pressure as above indicated.

The therapeutic cycle described was obtained following a 10 min incremental and gradual compression (from the atmospheric pressure to the therapeutic level), followed by an inversely similar 10 min period of decompression during which the subject breaths O2. No side effects after HBO2 were observed during the entire treatment.

**Fig. 2:**
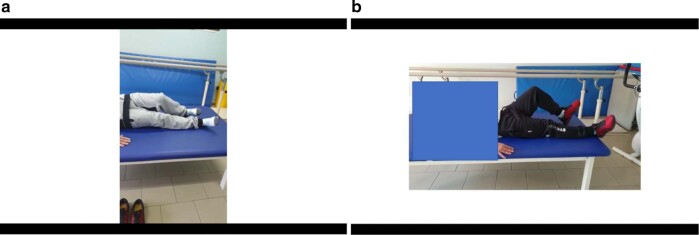
Photogram from the video recorded a week before before (**a**—left panel) and after (**b**—right panel) HBO cycle following the command to lift the left leg in eyes closed condition. **a** The frame shows the erroneous strategy used: the attempt was accomplished by large right to left shifts of both limb and trunk. **b** The correct movement of lifting in a straight line can be deduced by the position of the knee at 90° respect to both sides of the plane.

Regarding the clinical analysis, and in order to illustrate the main feature of the present study we show, before and after HBO2, two video frames (Fig. 2a, b) recorded during a particular exercise which epitomizes the peculiarity of the clinical features of this subject Briefly, while lying supine in the FKT table, in both conditions of eyes open and closed, the subject was required to slowly bend the legs, each in turn, having the knee constantly positioned at 90° respect the horizontal line of the table during the entire exercise. An eyes closed resting state EEG was recorded during the week preceding the HBO2 treatment 30 min before the above exercise and was followed by the same schedule 1 week after the HBO2 treatment.

For this study we use the portable wireless MUSE EEG system (InterAxon Inc., CAN) to collect data that would yield quantifiable EEG tracings [8] eventually available for a further study by the toolbox EEGLAB (2019 version). Briefly, in resting state conditions, we recorded two sessions of data, a week before and a week after HBO, from a MUSE EEG headband with preset AD at 256 Hz sampling rate (http://developer.choosemuse.com/ hardware- firmware/hardware) specifications for full technical information). The MUSE EEG system has electrodes located analogous to Fpz, AF7, AF8, TP9, and TP10 with electrode Fpz utilized as the reference electrode. The data obtained were streamed from the MUSE EEG system to MATLAB EEGLAB and read by an in-built EEGLAB program. From about 30 min of continuous recording we select for each study an artifact free part (6.000 ms), which is a time length optimally suited for estimate functional connectivity [9]. An independent component analysis (ICA) of the selected EEG recording was interpolated with the spherical splines which allow for the tri-dimensional location of the dipole generated by each electrode location by using the EEG LAB plugin FIELDTRIP and SIFT [10], the latter in order to implement the study of the “effective” connectivity intended as the influence one neural system exerts over another [11]. In order to obtain validated data accordingly, we used the normalized directed transfer function (DTF), a variant of the Granger causality algorithm which assumes that the connectivity information in a cause’s past “must improve the prediction of the effect above and beyond the information contained in the collective past “ of all other measured variables [10]. In its short math procedure, the process is expressed as ***ij (f)*** **=** ***k*** **=** ***1, Hij (f) ∑M Hik (f)2 0*** **≤** ***γij (f)2*** **≤** ***1 ∑M 2, j*** **=** ***1γ(f)*** **=** ***1 ij****,* which can be interpreted as the total information flow from j to i normalized by the total amount of information inflow to i. Generally, the magnitude- squared DTF **γij (f)2** is used [11]. The data obtained can also be graphically rendered by color-coded symbols during the time course selected either by a connectivity matrix or by the simulated brain visualization of the asymmetry index between the hub and edges (red maximum, blue minimum) by a direct graph projected in a three D template brain model.

## Discussion

**Fig. 3:**
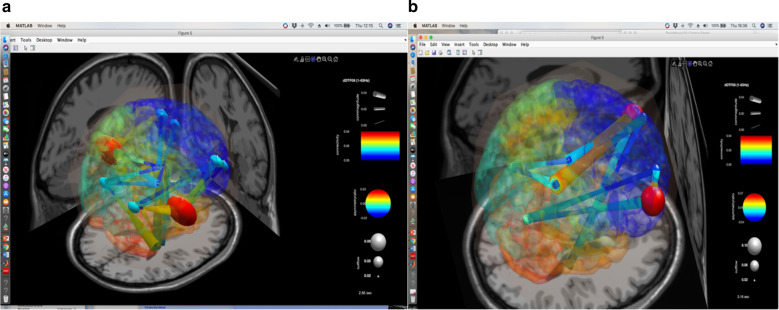
SIFT/Granger causality baseline for four component dipoles localized to the right, left frontal gyrus and the temporal–parietal regions Smaller, dark blue circles and dark blue, thin lines indicate lower causality, while red indicate high causality. **a** (left panel) Causality image at baseline occurring at 2850 of 6000 ms of EEG recording a week before HBO treatment. The effective connectivity (high causality) was depicted by the color-coded (full red circle) asymmetry index in the parietal-temporal hub and by the yellow-orange color of the direction of the edges originated from these areas. **b** (right panel). Causality image after a week from the completion of HBO cycle occurring at 3160 of 6000 ms. of EEG recording. The effective connectivity rendering is represented by the anterior full red circle which correlates with the asymmetry index in the frontal hub and by the origin of the yellow-orange color of direction of the edges originated from these regions.

Before HBO2 treatment, during the condition of eyes open the exercise of bending slowly the limbs was carried out properly, while during eyes closed condition the subject failed to perform the correct exercise as he tried to lift the leg by “flipping” the limb in a kind of irregularly oscillating lift (Fig. 2a: photogram from a short tape). However, a week after the HBO2 treatment the subject was able to perform the correct exercise as he followed a straight line while raising the leg (Fig. 2b: photogram). The results of the study of the effective connectivity before and after HBO2 are illustrated. The Fig. 3a (before HBO2) shows the prevalent effective connectivity as a temporo–parietal high asymmetry index directed toward the frontal region, represented by the large red colored spheres that express the dominance of the first cortical hub over the latter. Moreover, it is worth noting that the temporo–parietal area behaves as a hub which in turn give raise to robust edges (orange lines) equally directed toward the frontal regions. The Fig. 3b depicted the situation of the connectivity a week after HBO2. During this interval, which was considered according to the best FKT performances, the frontal areas now become the dominant collective hub-and-edges complex of an effective connectivity toward the temporo–parietal regions.

The results obtained show that the improved clinical conditions of this subject after HBO2 are correlated with a dramatic reshape of the hierarchical framework of the frontal-temporal–parietal connectivity compared to that showed before the treatment. Moreover, It is worthy of mention that the modular frontoparietal connectivity is highly integrated with the chain of movement effectors [12] as it instantiates any new task state by flexibly interacting with other control circuitries, such as the networks located in the spinal cord. Furthermore, the role that the frontal cortex plays in complex action timing, by planning and even in the orienting movements [13] suggests a bottom-up coherence in the hierarchical order of this circuitry. Finally, in considering such a choreography, it is of relevance to mention the crucial role played by the spinal interneurons in interacting with motor and sensory pathways of the spinal cord [14]. Though we cannot ascribe the modification in cortical connectivity as effect-or-epiphenomenon of the HBO2 treatment, the remodulation of the connectivity inside the frontal–temporo–parietal areas shows a close temporal correlation with HBO2 cycles. Although it is difficult to consider a single mechanism of action of HBO2 as the main promoter of the results obtained, the unchanged MRI features suggest that HBO2 effects are likely induced through other mechanisms. It has been suggested, for instance, that the action of HBO2 could be induced by several different agents, such as the reduced apoptosis, the decreasing of oxidative stress and the increasing of angiogenesis [4]. Moreover, in human subjects affected by traumatic brain injuries, a DTI MRI investigation shows that HBO2 improves brain connectivity by increasing the mechanism of neuroplasticity in the lesioned areas [15]. The present results suggest that the abnormal frontal–temporo–parietal connectivity observed before HBO2 can be consistent with the rupture of the regional modularity with the global hierarchical organization, and that the change occurring in these areas would represent an inefficient network “usage”. In this perspective these results could suggest that, before HBO, the temporal–parietal part of the neural circuitry is arbitrarily acting as a whole by downplaying the role of the frontal regions in the process of sensory-motor integration which instead should have been working in a flexibly interacting reciprocity [12]. Taken together, the crucial role of HBO2 in restoring the connectivity of the frontal areas could be part of a positive bottom-up feedback which can be hypothesized as starting from the improvement of the spinal interneuronal network, which is considered a critical structure in the mechanism of integration between motor and sensitive spinal neurons [14]. Following this suggestion, it seems likely that HBO2, by improving the role of the frontal areas in targeting the spinal cord neurons and by restoring/reshaping the global intercortical connectivity, might help in tuning the entire circuitry for better addressing the correct wiring pressure toward the spinal cord networks.

The improvement of the clinical symptoms after HBO2 described in this subject suggests that a rational approach in discussing the results obtained by using this treatment should take into account not only the spinal cord functions, but also the possible effects on their possible cortical correlations. However, while attempting to describe the changes of cortical connectivity before and after HBO2, this study deserves a critical note as the estimated connectivity has been incompletely defined, given the paucity of the electrode arrays used. In addition, a second cautionary note concerns the impact of the term “effective connectivity” exploited by the Granger causality, as this method cannot take into account the vector changing inside a cortical area which is deduced on the basis of an artifactual stationary configuration of the EEG signals, that instead are composed by a highly multidimensional continuous dynamic interplay of rhythms. Together, while taking into account these cautionary notes and though further investigations are need, this study suggests the useful of a more holistic approach in investigating the mechanism of action of HBO2 and could add a possible novel tool in the clinical monitoring of SCIs treatment.
